# A role for primary cilia in coral calcification?

**DOI:** 10.1007/s00441-020-03343-1

**Published:** 2020-12-16

**Authors:** Eric Tambutté, Philippe Ganot, Alexander A Venn, Sylvie Tambutté

**Affiliations:** Marine Biology Department, Centre Scientifique de Monaco, 8 Quai Antoine 1°, 98000 Monaco, Monaco

**Keywords:** Cilium, Biomineralization, Acetylated tubulin, Scanning electron microscopy, Calicoblastic ectoderm

## Abstract

Cilia are evolutionarily conserved organelles that extend from the surface of cells and are found in diverse organisms from protozoans to multicellular organisms. Motile cilia play various biological functions by their beating motion, including mixing fluids and transporting food particles. Non-motile cilia act as sensors that signal cells about their microenvironment. In corals, cilia have been described in some of the cell layers but never in the calcifying epithelium, which is responsible for skeleton formation. In the present study, we used scanning electron microscopy and immunolabelling to investigate the cellular ciliature of the different tissue layers of the coral *Stylophora pistillata*, with a focus on the calcifying calicoblastic ectoderm. We show that the cilium of the calcifying cells is different from the cilium of the other cell layers. It is much shorter, and more importantly, its base is structurally distinct from the base observed in cilia of the other tissue layers. Based on these structural observations, we conclude that the cilium of the calcifying cells is a primary cilium. From what is known in other organisms, primary cilia are sensors that signal cells about their microenvironment. We discuss the implications of the presence of a primary cilium in the calcifying epithelium for our understanding of the cellular physiology driving coral calcification and its environmental sensitivity.

## Introduction

Cilia, or flagella, are evolutionarily conserved organelles that extend from the surface of cells and exist in diverse organisms from protozoans to multicellular organisms (Davis et al. 2006; Singla and Reiter 2006; Song et al. 2016). The structure of the cilium is based on the axoneme, a columnar array of microtubules that are templated directly by the mother centriole of the centrosome, often termed the basal body. Cilia can be classified into groups by whether they have a 9 + 2 or a 9 + 0 arrangement of axonemal microtubules and by the presence or absence of motility. Although assigned different names to reflect their different beating motions, cilia and flagella are structurally similar (the two names are sometimes used interchangeably (Singla and Reiter 2006), but here, we will use the term cilia). Generally speaking, 9 + 2 structures are motile and can be present as numerous cilia on the cell surface, and 9 + 0 structures are not motile and occur as a monocillium on the cell surface, though this is not a hard and fast rule (Bloodgood 2009). Establishing the link between the axonemal microtubule structure, the motility of cilia and the number of cilia per cell is thus quite tricky. In addition to the axonemal microtubule structure, cilia also show diverse structural features such as shape and length (Eley et al. 2005). In vertebrates, cilia are present in nearly every cell type and perform diverse biological functions. Motile cilia are involved in mixing fluid or keeping the airways clear of mucus and dirt such as in the pulmonary track. Non-motile cilia, also called primary cilia, were previously considered as “vestigial organelles” but are now viewed as sensors that further signal cells about their microenvironment (Alaiwi et al. 2009; Pazour and Witma 2003). Defects in primary cilia are observed in human diseases (called “ciliopathies”) characterized by many disorders such as kidney and liver diseases, vision loss, obesity, diabetes and cancers (Fabbri et al. 2020; Ware et al. 2011).

In invertebrates, cilia also perform diverse biological functions. For example in turbellarians or in gastropods, ciliary gliding allows a type of locomotion in which the animal is propelled by the beating of cilia on a secreted layer of mucus (Martin 1978). In filter-feeding organisms such as in brachiopods, cilia catch particles and transfer them to the mouth (Riisgard and Larsen 2001; Riisgård and Larsen 2010). Cilia have also been observed in stony corals and sea anemones (Phylum: Cnidaria, Class: Anthozoa, Subclass Hexacorallia). These animals show a simple anatomy and histology: their anatomical unit is a polyp consisting of a vase-shaped body with a central mouth surrounded by a ring of tentacles and a column as the main body. The tissues consist of two epithelial layers named epidermis and gastrodermis, referring to the adult epithelia, or ectoderm and endoderm, referring to their embryological origins. Since in the literature, ectoderm and endoderm are also classically used for coral adult epithelia, we will use these terms in this paper. The ectoderm and the endoderm are connected by an extracellular matrix called mesoglea. Uniformly arranged cilia cover the oral ectoderm that faces seawater and is surrounded by a mucus layer (Brown and Bythell 2005). Cilia beating gives corals the ability to vigorously stir the water at the coral surface, allowing control of the exchange of nutrients and oxygen between the coral and its environment by enhancing mixing in the so-called diffusive boundary layer (Pacherres et al. 2020; Shapiro et al. 2014). Cilia of the oral endoderm are also directly involved in feeding in several respects. Firstly, the presence of a multicellular structure called the ciliary cone, on the stinging cells, nematocytes, at the tentacle tips (acrospheres), suggests that a complex of cilia and microvilli could be involved in mechanoreception and nematocyst discharge (Fautin and Mariscal 1991). Secondly, mucociliary transport of food particles, trapped by the mucous surface, occurs to the mouth of the coral polyp and then into the coelenteron (body cavity), where they are digested (Brown and Bythell 2005). However, cilia are not restricted to the oral ectoderm and the endoderm that lines the coelenteron is also covered by cilia (also described as flagella (Eppard et al. 1989)). In symbiotic corals that contain photosynthetic dinoflagellates in their endodermal cells, in addition to moving fluid and food within the coelenteron cavity, these cilia produce currents that may enhance acquisition of symbionts by late developmental stage larvae (Harii et al. 2009) and in fast growing zones in adults (pers.obs).

Whereas cilia have been described in the ectoderm facing seawater and the endoderms facing the coelenteron, to our knowledge, no cilia have been reported in the calicoblastic ectoderm that contains the calcifying cells responsible for coral skeleton formation. These calcifying cells control the composition of a microenvironment named the extracellular calcifying medium (ECM (Tambutté et al. 2011)), at the interface between themselves and the skeleton surface. This biological control by the cells involves (i) the regulation of the ionic composition of the ECM including pH, carbonate and calcium concentration (Sevilgen et al. 2019; Venn et al. 2011); (ii) the exocytosis of organic matrix molecules (Puverel et al. 2005) and amorphous calcium carbonate nanoparticles (Mass et al. 2017).

Since calcification is a major and essential process at the basis of the foundation of coral reef ecosystems, the biological control of calcification has been the subject of many physiological, biochemical and molecular studies (reviewed in Drake et al. 2020; Tambutté et al. 2011). However, despite the widely recognized ecological importance of coral reef ecosystems and their reliance of coral calcification to provide their structure, knowledge of the cellular aspects of coral calcification is still relatively scarce, especially about how cells sense the extracellular environment (Ganot et al. 2020). In humans, it is known that cilia play a key role in chondrogenesis and skeleton formation where they act as an antenna that senses extracellular signals (Moore and Jacobs 2018; Olsen 2005). Cilia and cilia-related proteins are involved in osteoblast differentiation and mechanical stimulation-induced osteogenesis and ciliary defects result in severe skeletal abnormalities, confirming the indispensable role of cilia in bone development and homeostasis (Yuan et al. 2015). Based on the fundamental role that cilia play in skeleton formation in vertebrates, we asked whether cilia could be present in coral calcifying cells. We used scanning electron microscopy and confocal microscopy immunolabelling with anti-acetylated-tubulin and phalloidin to investigate the cellular ciliature of the different tissue layers composing the coral *Stylophora pistillata*, with a special focus on the calcifying calicoblastic ectoderm.

## Material and methods

### Biological material

All experiments were conducted on samples prepared from colonies of *Stylophora pistillata* maintained at the Centre Scientifique de Monaco. Samples were prepared by the lateral skeleton preparative assay (Muscatine et al. 1997; Raz-Bahat et al. 2006; Venn et al. 2011). Briefly, microcolonies of *S. pistillata* were allowed to rest on glass slides so that the basal portion of the colony grew out over the slide as a thin sheet. Pieces of sheets were sectioned from the colonies with a razor blade and fixed with resin (DevconTM) to glass slide. Pieces of corals were then left to grow out across glass slide in aquariums (these were the samples used for experiments) supplied with flowing seawater from the Mediterranean Sea (exchange rate 2% h^−1^) at a salinity of 38, a temperature of 25 °C, under irradiance of 170 mmol photons m^−2^ s^−1^ on a 12 h/12 h photoperiod. Algae were periodically removed from the glass coverslip by a razorblade.

## Field emission scanning electron microscopy

Samples (*n* = 10) were processed as described in (Tambutté et al.  2011). Briefly, samples were fixed overnight at 4 °C with 4% glutaraldehyde in 0.085 M Sorensen phosphate buffer at pH 7.8 with 0.5 M sucrose. Decalcification was achieved by transferring the samples to a mixture of 0.085 M Sorensen phosphate buffer, 0.5 M sucrose containing 2% glutaraldehyde and 0.5 M ethylenediaminetetraacetic acid (EDTA) at pH 7.8 and 4 °C. This solution was renewed until decalcification was completed. Decalcified samples were rinsed in Sorensen buffer, then post-fixed for 1 h at ambient temperature with 1% osmium tetroxide in Sorensen phosphate buffer. Samples were dehydrated by transfer through a graded series of ethanol ending with a concentration of 100%. After dehydration, they were incubated for 15 min in hexamethyldisilazane (HMDS)/ethanol 100% (*v*/*v*), then 30 min in HMDS 100% that was subsequently evaporated under a fume hood overnight. Samples were then coated with gold-palladium and observed at 3–5 kV with a JEOL JSM-6010LV.

## Immunolabelling

Samples (*n* = 7) were processed as described in (Ganot et al. 2020). Briefly, samples were fixed in 50-ml chilled artificial-sea-water/paraformaldehyde (PAF) fixation buffer (425 mM NaCl, 9 mM KCl, 9.3 mM CaCl2, 25.5 mM MgSO4, 23 mM MgCl2, 2 mM NaHCO3, 100 mM HEPES pH 7.9, 4.5% PAF) for 2–4 h at 4 °C. Samples were decalcified in 50 ml (100 mM HEPES pH 7.9, 500 mM NaCl, 250 mM EDTA pH 8.0, 0.4% PAF) at 4 °C for 3 days, washed 3 times with PBST (1× PBS, 0.1% Tween_20), then blocked in (1× PBS, 2% BSA, 2% donkey serum, 0.1% Triton_X100) for 2 h at 4 °C. Samples were further incubated in the blocking solution complemented with AlexaFluor_488 phalloidin (A12379, ThermoFisher) and the following antibodies: primary antibody solution/mouse anti-acetylated tubulin (ab24610, Abcam) 1:250 for 2 days at 4 °C; secondary antibody solution: Goat anti-Mouse AlexaFluor_555 (A32727, ThermoFisher) 1:500 for 2 days at 4 °C. Finally, samples were rinsed three times for 10 min in PBST containing DAPI and mounted on glass coverslips in Slowfade Gold antifading agent (ThermoFisher). Controls were routinely performed with only the secondary antibody and, with similar settings as in experiments with primary antibody, no signal was observed. Imaging was performed using a confocal Leica SP8 and the LASX lite software. For imaging, each channel was acquired sequentially.

## Results

**Fig. 1:**
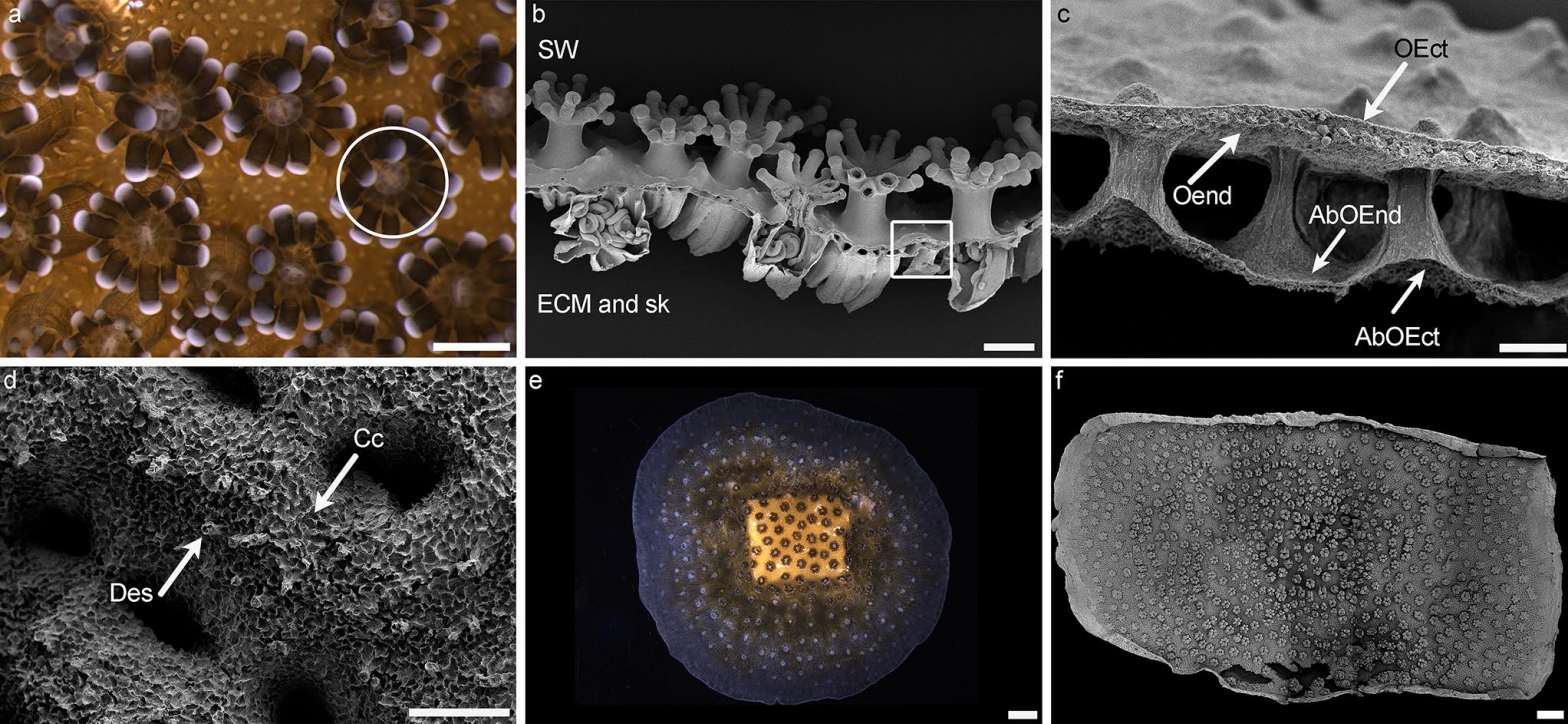
Anatomy and histology of *Stylophora pistillata*. **a** Living colony observed showing polyps with their expanded tentacles and their mouth (circle around one polyp, Leica Macrofluo observation). **b** Decalcified fixed colony showing polyps linked together by the coenosarc (square shows the coenosarc, scanning electron microscopy image). **c** Zoom of the coenosarc in **b** showing the oral and aboral tissues with ectoderms and endoderms. **d** Zoom of the calicoblastic ectoderm (observation of side facing the skeleton) showing desmocytes and calicoblast calcifying cells. **e** Observation of a living coral fragment growing on slide according to the lateral preparative assay, observation of the side facing seawater (image of sample under bright light observed under a binocular). **f** Observation of a decalcified coral fragment growing on slide according to the lateral preparative assay, observation of the side facing the glass slide (scanning electron microscopy image). SW = seawater side. sk = skeleton side. ECM = extracellular calcifying medium side. OEct = oral ectoderm. Oend = oral endoderm. AbOend = aboral endoderm. AbOect = aboral ectoderm = Calicoblastic ectoderm. Des = desmocytes, Cc = calcifying calicoblast cells. Scale bars: **a** = 1 mm, **b** = 500 µm, **c** = 50 µm, **d** = 50 µm, **e** = 3 mm, **f** = 3 mm

*Stylophora pistillata* possesses the typical anatomy of colonial scleractinian corals with polyps linked together by the cœnosarc (Fig. 1 a-c). The histology consists of an oral tissue facing seawater and an aboral tissue (Fig. 1c) each being composed of an ectoderm separated from the endoderm by a connective layer of mesoglea (mostly composed of collagen fibres and water). The aboral ectoderm (or calicoblastic ectoderm (Johnston 1980)) is composed of the calcifying cells, so-called “calicoblast cells” (Johnston 1980), and the desmocytes (Muscatine et al. 1997) which anchor the tissue to the skeleton and do not calcify (Fig. 1d). For the present work, in order to avoid long decalcification steps of our samples, we choose to work with samples grown according to the lateral skeleton preparative assay (Muscatine et al. 1997; Raz-Bahat et al. 2006; Venn et al. 2011) where coral fragments directly grow on glass slides (Fig. 1e, f) and deposit crystals that will then form the skeleton.

**Fig. 2:**
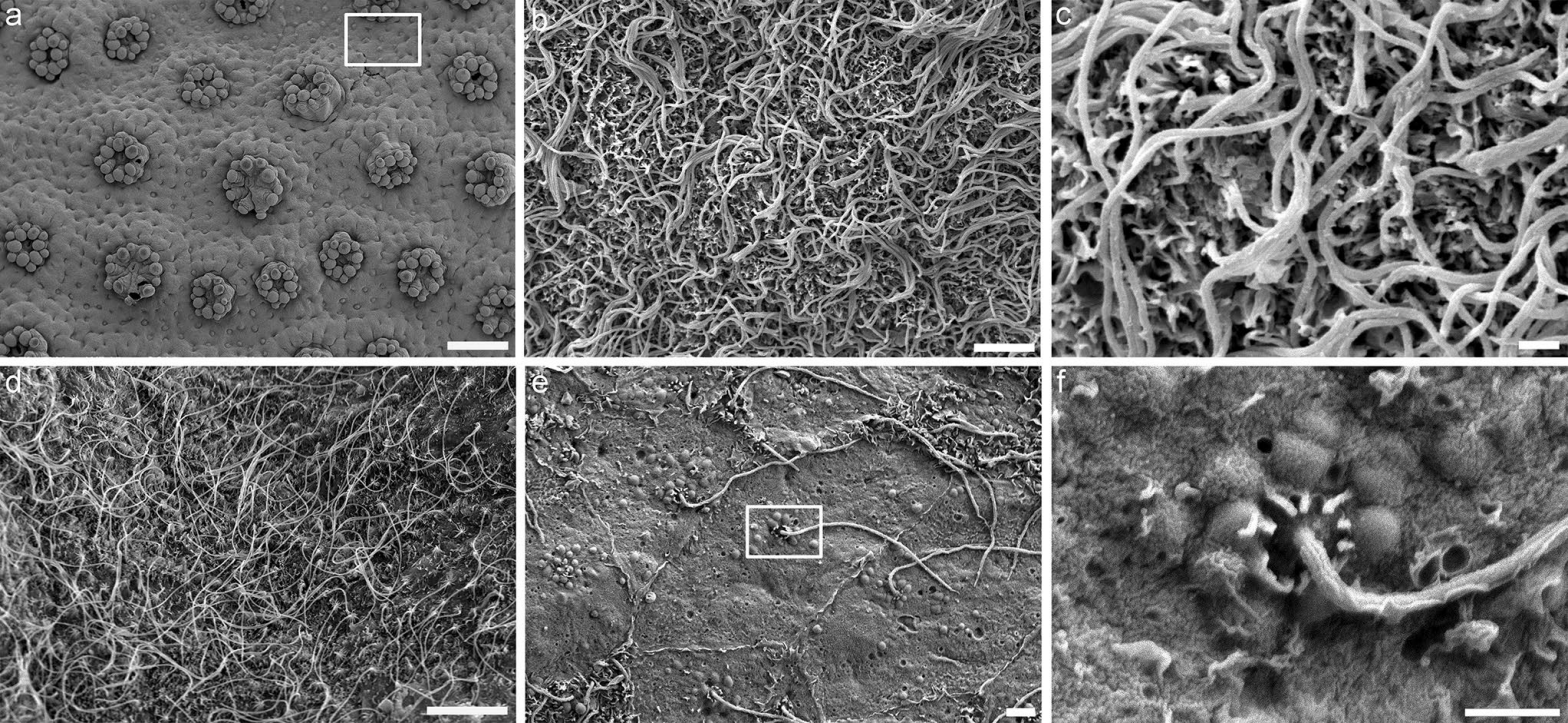
Oral ectoderm of *Stylophora pistillata* observed with the scanning electron microscope. **a** Overview of the oral ectoderm with polyps and coenosarc (square shows the coenosarc). **b**, **c** Zoom of the coenosarc in **a** showing a dense array of long cilia. **d** Other region of oral ectoderm with dense cilia showing “flower-like pattern” at the base. **e** In some regions, the cilia are less dense. **f** Magnification of square in **e** showing «flower like» pattern at the base of the cilia. Scale bars: **a** = 500 µm, **b** = 10 µm, **c** = 5 µm, **d** = 1 µm, **e** = 2 µm, **f** = 1 µm

**Fig. 3:**
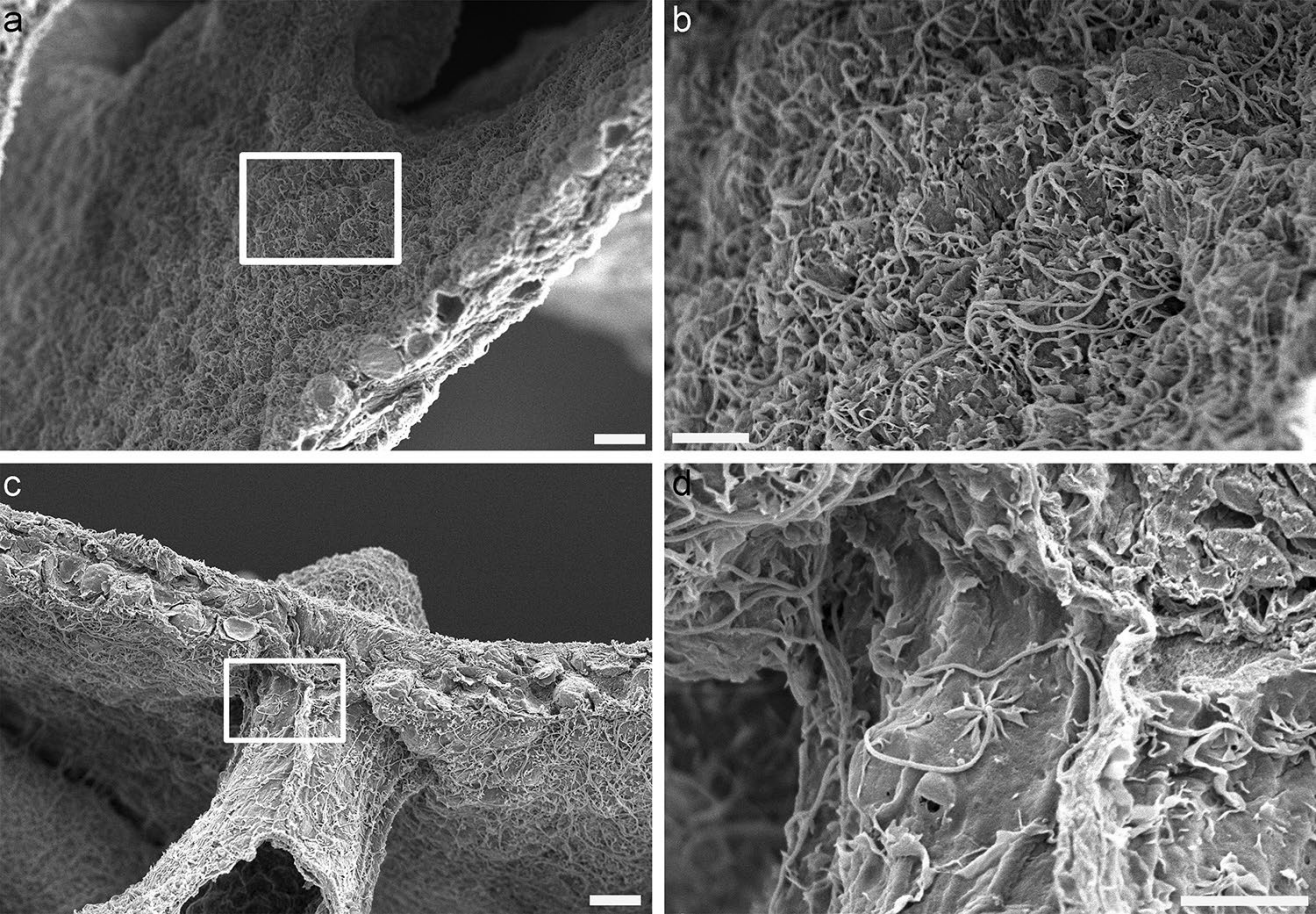
Oral endoderm of *Stylophora pistillata* observed with the scanning electron microscope. **a** Overview of the oral endoderm. **b** Magnification of square in **a**, showing a dense array of long cilia. **c** Other regions of endoderm where cilia are more or less dense. **d** Magnification of square in **c** where a «flower like» pattern can be observed at the base of the cilium. Scale bars **a** = 10 µm, **b** = 10 µm, **c** = 5 µm, **d** = 5 µm, **e** = 20 µm, **f** = 5 µm

**Fig. 4:**
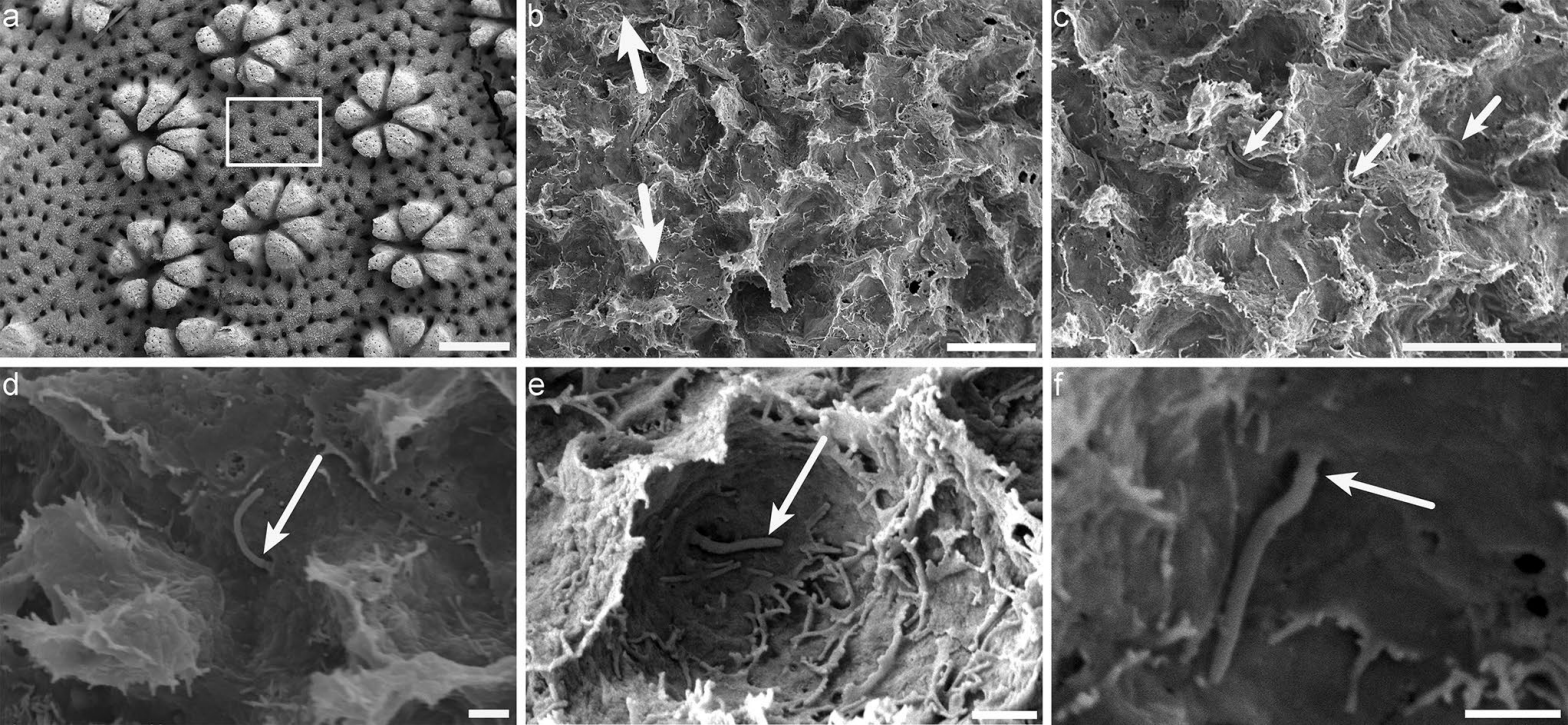
Aboral calicoblastic ectoderm observed with the scanning electron microscope. **a** Overview of the aboral ectoderm. **b–f** Magnifications of aboral ectoderm (square in **a**), showing that almost all calicoblast cells possess a short cilium (arrow). Note that there is one cilium in each calcifying cell. No «flower-like» pattern is observed at the base of the cilium, but instead, there is a ciliary pocket (arrow in **f**). Scale bars **a** = 500 µm, **b** = 10 µm, **c** = 5 µm, **d** = 1 µm, **e** = 1 µm, **f** = 1 µm

**Fig. 5:**
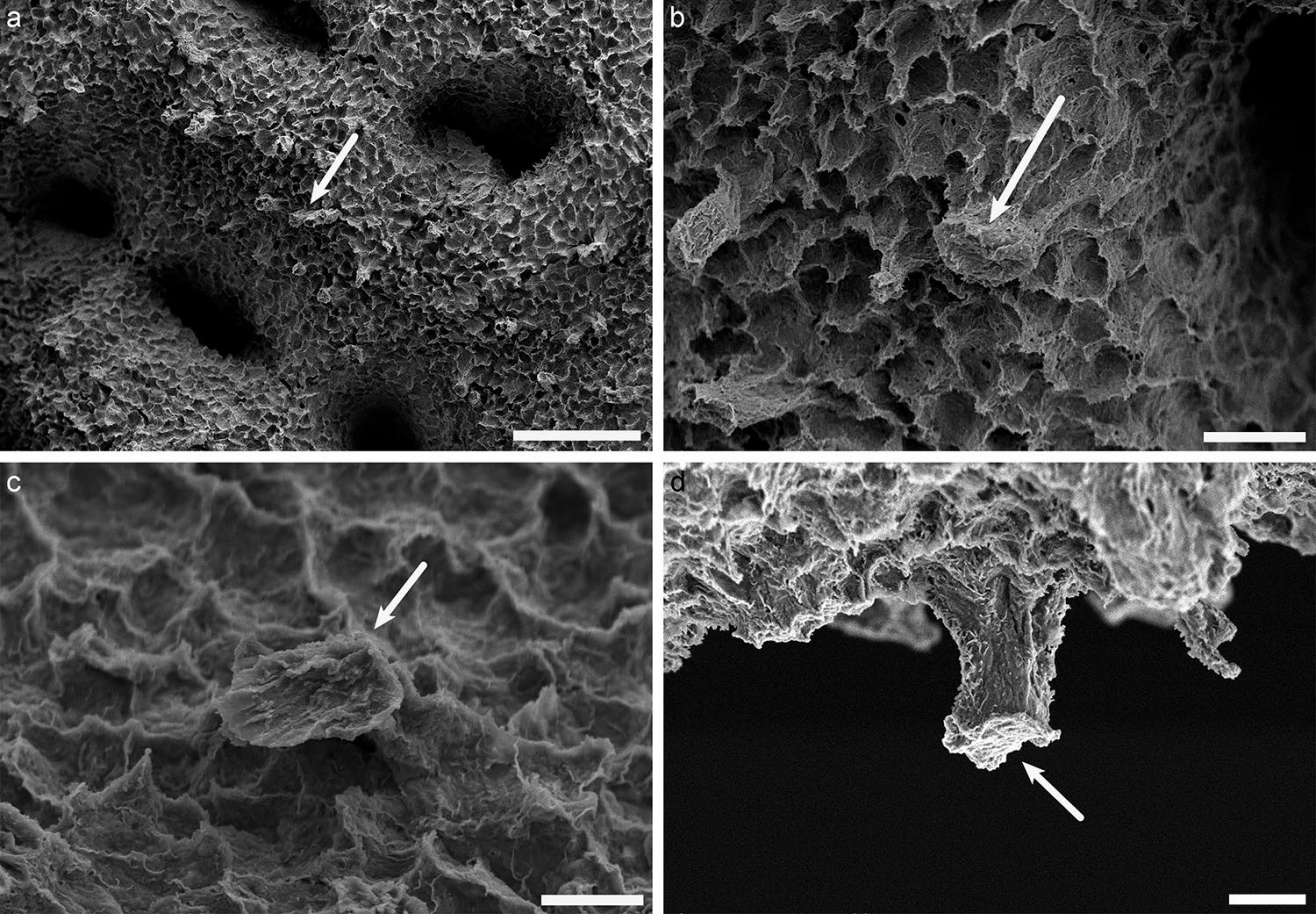
Aboral calicoblastic ectoderm observed with the scanning electron microscope. **a** Overview of the aboral ectoderm. **b**–**d** Magnification of **a**, showing that desmocytes (arrow), which anchor the epithelium to the skeleton but do not calcify, do not possess a cilium. Scale bars **a** = 50 µm, **b** = 10 µm, **c** = 5 µm, **d** = 5 µm

Using scanning electron microscopy, we observed two different types of cilia structure in the tissue layers. Firstly, in the oral ectoderm as well as in the endoderms (Figs. 2 and 3; Fig. S2), a single long (in the 10 to 20 µm range) cilium was observed on a large majority of cells. Ciliary cones were observed on tentacle tips (acrospheres) of the oral ectoderm (Fig. S1). The density of cilia varies from low to high on the oral ectoderm and endoderms (Figs. 2 and 3). At the base of the cilium, both in oral ectoderm and endoderms, a “flower like” pattern was observed (Figs. 2d–f and 3d). Similar pattern was already described in other anthozoan species (Fautin and Mariscal 1991). This cilium structure was strikingly different to the structure of the second type of cilia observed on the calicoblast cells (Fig. 4; Fig. S3). These cells possessed a single, very short (1 to 2 µm) monocillium emerging strait from the apical cell membrane (Fig. 4c–f; Fig. S3d, S3e) and in some cases the presence of an invagination of the membrane called a “ciliary pocket” (Chang et al. 2015) at the base of the cilium (Fig. 4f). As a rule, we never observed more than one cilium per cell. Monocilia were absent on certain cells in the calicoblastic epithelium, and they were consistently absent on the desmocyte subtype (Fig. 5). In addition to these observations performed on tissues overlying mature skeleton, we also observed a zone where calicoblast cells form new calcium carbonate crystals in the early steps of calcification (Fig. S3). In this zone, a monocillium was present on each calcifying cell (Fig. S3d, e). Despite differences in cell shape and density, we found no differences in cilium structure regardless of the zone of calcifying cells that we observed (i.e., over newly forming skeleton or different areas of mature skeleton).

**Fig. 6:**
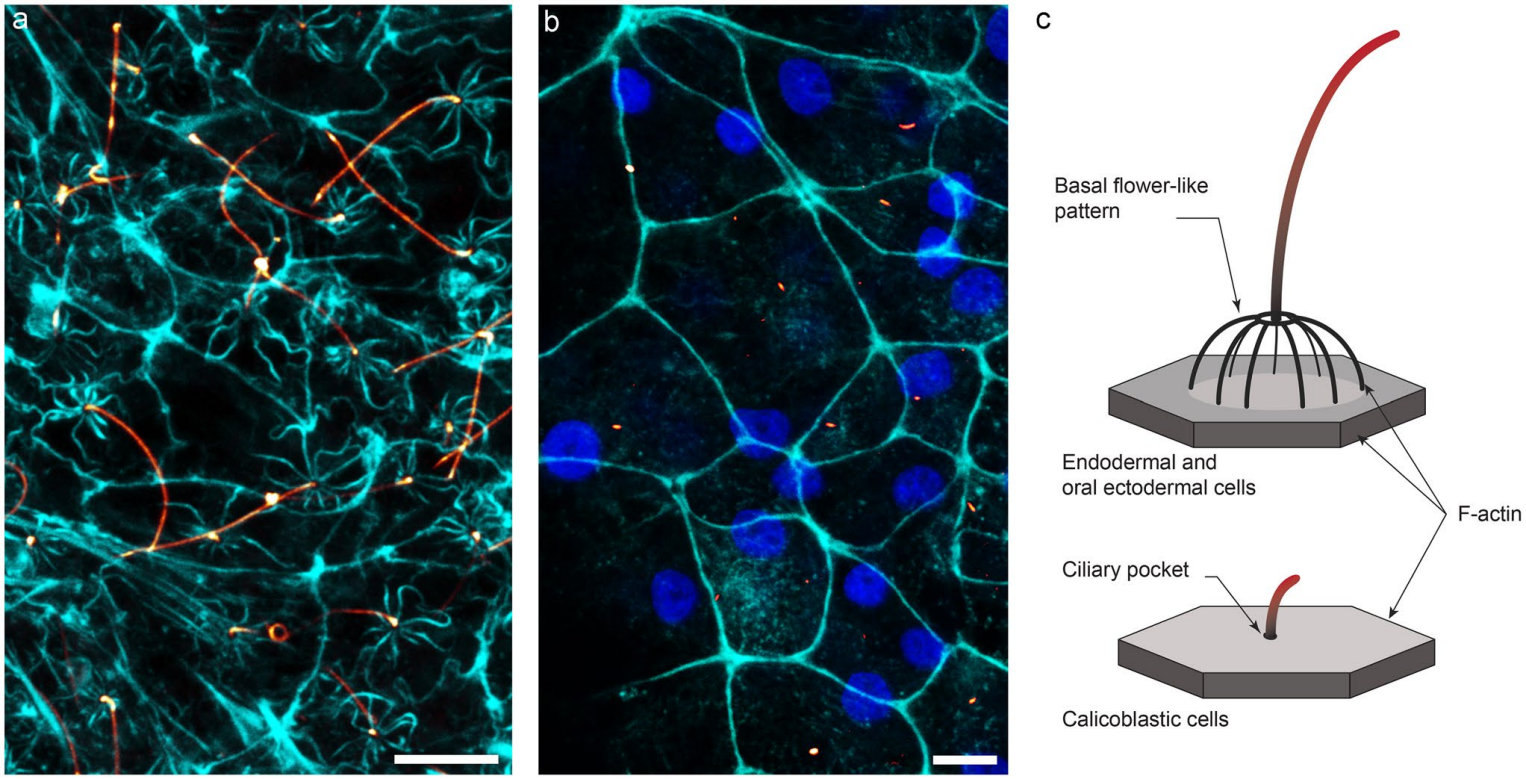
Structure of the two different types of cilia. **a** Aboral endoderm and **b** aboral ectoderm are confocal image stacks of the cells after immunolabelling with anti-acetylated tubulin (glow), phalloidin (cyan) and DAPI (blue). Note the radiating actin labelling emanating at the base of cilia with a “flower-like” pattern in **a** that is completely absent in calicoblast cells **b**. **c** schematic structure of the cilia present in the 3 tissue layers involved in fluid movements (the boundary layer for the oral ectoderm, and the two endoderms lining the coelenteron) and the “primary cilia” type (which are on the calcifying cells in contact with the ECM). Axoneme, F-actin and plasma membrane are represented in red, black and grey, respectively. Scale bars **a**, **b** = 5 µm

Since the main structural component of axonemes are polymers of modified tubulin, mostly acetylated tubulin, we used immunofluorescence with anti-acetylated-tubulin to image, using confocal microscopy, cilia of the different *Stylophora pistillata* cell layers. Co-staining with phalloidin allowed visualization of the F-actin network delineating the epithelial apical cell borders (Fig. 6 and tridimensional reconstruction videos, Suppl. Videos 1 and 2). At the base of the cilium in endoderm, we observed the same “flower-like pattern” as we observed with SEM (Fig. 6a; Suppl. Video 1). This protuberant base was composed of actin rays radiating around the cilium. In the cases we observed, we counted nine rays of actin. Consistent with our SEM observations, this cilia structure was different from the structure of the cilia observed for the calicoblast cells. The monocillium observed on the calicoblast cells was shorter and lacked the “flower-like” pattern at the base (Fig. 6b; Suppl. Video 2). Instead, it emerges straight from the apical cell membrane like an “antenna”.

Thus, in *Stylophora pistillata*, as with scanning electron microscopy, two types of acetylated tubulin containing cilia were observed: the long cilia of the endoderms and oral ectoderm and the newly identified short cilia observed on the calicoblast cells. A schematic representation of the two types of cilia is presented in Fig. 6c.

## Discussion

In the present study, we show that cilia exist in all cell layers of the reef-building model coral *Stylophora pistillata*. The cilia observed in three of the four coral epithelial layers, the oral ectoderm, and the endoderms correspond to the cilia previously described in hexacorallians (Fautin and Mariscal 1991; Shapiro et al. 2014). As described in the introduction, these cilia have been shown to be motile and play a role in moving fluid and transporting food. In the present study, we reveal for the first time that cilia are also present in the calicoblast calcifying cells.

The cilium of the calicoblast cells in the aboral ectoderm is a monocillium that shows differences with the motile cilia of the other epithelial cell layers: it is much shorter and immunolabelling shows that this monocillium emerges straight from the apical cell membrane from within a ciliary pocket as opposed to the “flower-like” pattern at the base for cilia observed in the other tissue layers. Based on these observations and by comparison with the data in the literature for vertebrates (for scanning electron microscopy observations, see for example Fig. 1a in Chang et al. (2015), Fig. 2 in Benmerah et al. (2015), Fig. 3 a in Liem Jr et al. (2012) and for immunolabelling with anti-acetylated tubulin see for example Fig. 4 a in Clement et al. (2009)), our results lead us to conclude that the cilium of the calicoblast cells is a primary cilium.

Using this finding as starting point, our aim is to discuss the implications of the presence of a primary cilium in the calicoblast ectodermal cells for our understanding of coral calcification. Based on what is known in vertebrates, we raise a number of questions in corals that we believe could open exciting new avenues of research for cell biologists and physiologists working on both cell biology and calcification.

The presence of a primary cilium raises a number of questions for coral biology and particularly calcification that start with the topic of ciliogenesis (i.e. the timing of primary cilium formation). In mammalian cells, the primary cilium structure is based on the axoneme (microtubule doublets) templated by the mother centriole of the centrosome that then forms the basal body (Nigg and Stearns 2011). Hence, the primary cilium does not exist when the centrosome is engaged in assisting mitosis. The mammalian centrosome duplicates during the S-phase of the cell cycle, and then organizes the tubulin-based mitotic spindle (G2-M). As such, it exits from its basal body function. Thus, the primary cilium exists only in post-mitotic cells, in differentiated proliferating cells during G1, or quiescence phases (G0). In other phyla such as insects (e.g. fruit flies), procentriole formation (daughter centriole initiation) starts already at telophase, and centrosomes are duplicated almost throughout the entire cell cycle. In these organisms, the primary cilium exists only in a small number of cell types, and in specific cell lineages, the two centrioles are able to promote formation of an axoneme (Lattao et al. 2017; Riparbelli et al. 2020). In anthozoans, the biology of centrioles and centrosomes along the cell cycle is not well documented; this is especially true for the calicoblast cells. Calicoblast cells number increases with colony growth and cell division figures can be observed at low rate (pers. obs), but little is known about their cell cycle, even less about their centrosomes. Clearly, some cells possess a primary cilium and others do not. We cannot rule out that the absence of cilia on some cells may represent technical artefacts due to the difficulty of preserving the complete cellular integrity of this rather inaccessible tissue layer, visible only after decalcification. However, the presence or absence of a monocillium may also reveal differences in cell cycle progression, senescence, differentiation stage, specific cell lineage and/or in function in regard to calcification, such as secretion of organic matrix proteins and control of the physico-chemical composition of the extracellular calcifying medium (ECM, which is the medium at the interface between the aboral calicoblastic ectoderm and the skeleton), among others. Indeed, it is noteworthy that the desmocytes (cells that anchor the epithelium to skeleton but do not calcify (Muscatine et al. 1997) do not possess a primary cilium. Future studies could thus investigate whether the presence of a monocillium can be used as a marker of differentiated cells within this calcifying ectoderm. Information could be derived from experiments performed during developmental stages, such as by comparing pre-calcifying larvae with settled calcifying larvae.

Next, we can ask about the role of this primary cilium in coral calcifying ectodermal cells. Indeed, whereas the primary cilia were first considered as a “vestigial organelles”, they are now regarded as having a pivotal role in transducing external cues (Song et al. 2018; Yuan et al. 2015). It has been shown that it is the bending of the primary cilium with fluid flow in the microenvironment that triggers the mechanical signal for mechanoreception. For example in kidney cells, the primary cilia respond to the mechanical signal produced by the flowing liquid (urine) to initiate rapid uptake of calcium, whereas cells lacking primary cilia show no response to flow (Singla and Reiter 2006). Several studies have provided strong evidence that primary cilia play a mechanosensory function not only in epithelial cells but also in bone cells (Nguyen and Jacobs 2013). However, in the case of bone, it is unclear whether there is enough space in the microenvironment between the cells and the bone for the primary cilium to bend with fluid flow (Temiyasathit and Jacobs 2010). This question is also very pertinent for corals. Indeed, estimates of the thickness of the ECM vary from approximately a few nanometers to more than 1 μm in width with possible diurnal variations (Tambutté et al. 2011). “Pockets” or “cavities” where the aboral ectoderm is lifted away from the skeletal surface have also been observed (Barnes 1970; Venn et al. 2011). Moreover, several studies have looked at the role of calcifying cell trans-membrane transporters in controlling the ECM physico-chemical composition (Bhattacharya et al. 2016; Zoccola et al. 2015, 2004, 1999), and one has considered how the calcifying cells sense the acid-base challenge of the external medium (Barott et al. 2020). To our knowledge, none has looked at other processes that can sense the physical or chemical properties of the ECM or skeleton. So, how the cell and its cilium senses the characteristics of the ECM/skeleton will really worth being considered in the future.

It is noteworthy that in bones, the primary cilium plays a role in signalling through the TGFB/BMP pathway (Clement et al. 2013). Indeed, receptors of bone morphogenetic proteins (BMP) localize to the primary cilium (Lindbaek et al. 2015). In corals, the presence of a BMP2/4 orthologue was reported in the eight species (Zoccola et al. 2009). Immunolabelling in *Stylophora pistillata* with an antibody against BMP shows a cytoplasmic and cell membrane labelling, indicating that these calcifying cells secrete BMP2/4 towards the skeleton. We can thus ask whether there is a link between BMP signalling pathway and the primary cilium in the coral calcifying cells, and if yes, whether this signalling pathway regulates calcification through the primary cilium or more broadly the physiology of the calcifying cells.

Finally, it would be interesting to relate ciliogenesis to the performance of calcification on different substrates or in different environments. Indeed, in vertebrate cultured cells, physical parameters such as spatial confinement and substrate rigidity, through their effect on actin cytoskeleton architecture, regulate ciliogenesis at cell cycle exit (Pitaval et al. 2010). When individual cells are sufficiently spatially confined on adhesive substrates, they can mimic an apico-basal polarity of epithelial cells and most of them assemble a primary cilium. Conversely, when individual cells are widely spread, there is no growth of the primary cilium (Pitaval et al. 2010). Moreover, a majority of cells grown on soft substrates (polyacrylamide gel grafted on glass coverslips) are ciliated cells, whereas only a few cells are ciliated when grown on a hard substrate (polystyrene-coated glass coverslips). Taking into account this observation, we can ask whether it is possible in corals, through testing different substrates that would manipulate ciliogenesis of the calcifying ectodermal cells, to see whether substrate characteristics affect cell polarity and the performance of calcification. Technically, this would be feasible by using the lateral preparative assay used in the current study where coral fragments expand with calcifying cells growing directly on glass slides. Such experiments can also be combined with experiments aimed at determining the impact of environmental stress on calcification such as ocean acidification (Gattuso and Hansson 2011) or ocean temperature increase (Bernardet et al. 2019) since for example in mammalian and zebrafish cells, it has been shown that primary cilia are sensitive to temperature stress (Prodromou et al. 2012). This would have implications for initiatives that are already being taken to preserve and restore coral reefs (Monty et al. 2006). Indeed, among the proposed ecological solutions of reef restoration, an important approach is the growth of corals in nurseries, before outplanting them on reefs in order to repopulate degraded areas. Observing the effect of substrates on ciliogenesis in corals and understanding its role in coral calcification could provide a laboratory-based approach to screening suitable substrates for coral recruitment and growth that could assist coral reef restoration strategies.

## Conclusion

In the present study, we showed that a monocillium with structural characteristics of a primary cilium is present in coral calcifying cells. Although a better understanding of the role of this cilium in coral calcification will probably await technical developments, our observations open new perspectives for research. For coral biology, future research on cilia may provide information about calcification, both at the fundamental level of the mechanistics of invertebrate calcification of this process and at the applied level of calcification performance on different substrates or under different environmental conditions. More generally, future research will also need to consider whether an animal as anatomically and histologically simple as a coral can serve as a model in the field of cilia biology. This new and simple model could be particularly useful in the study of the many ciliopathies related to cilia dysfunction.

## Electronic Supplementary Material

Below is the link to the electronic supplementary material.

Supplementary file1 (TIF 3.3 kb)

Below is the link to the electronic supplementary material.

Supplementary file2 (TIF 4.6 kb)

Below is the link to the electronic supplementary material.

Supplementary file3 (TIF 4.8 kb)

Below is the link to the electronic supplementary material.

Supplementary file4 (MP4 12.7 kb)

Below is the link to the electronic supplementary material.

Supplementary file5 (MP4 4 kb)

## Supplementary information

The online version of this article (10.1007/s00441-020-03343-1)

## References

[CR1] Alaiwi WAA, Lo ST, Nauli SM (2009). Primary cilia: Highly sophisticated biological sensors. Sensors.

[CR2] Barnes DJ (1970). Coral skeletons: An explanation of their growth and structure. Science.

[CR3] Barott KL, Venn AA, Thies AB, Tambutté S, Tresguerres M (2020). Regulation of coral calcification by the acid-base sensing enzyme soluble adenylyl cyclase. Biochem Biophys Res Commun.

[CR4] Benmerah AB et al (2015) The more we know, the more we have to discover: An exciting future for understanding cilia and ciliopathies. *Cilia*:1–13 10.1186/s13630-015-0014-0 PMC4378380 25974046

[CR5] Bernardet C, Tambutté E, Técher N, Tambutté S, Venn AA (2019). Ion transporter gene expression is linked to the thermal sensitivity of calcification in the reef coral Stylophora pistillata. Scientific Reports.

[CR6] Bhattacharya D (2016). Comparative genomics explains the evolutionary success of reef-forming corals. Elife.

[CR7] Bloodgood RA (2009) From Central to Rudimentary to Primary: The History of an Underappreciated Organelle Whose Time Has Come. The Primary Cilium. *In* Methods in Cell Biology, vol 94. Elsevier 2–52 10.1016/S0091-679X(08)94001-2 20362083

[CR8] Brown BE, Bythell JC (2005). Perspectives on mucus secretion in reef corals. Mar Ecol Prog Ser.

[CR9] Chang C-F, Schock EN, Attia AC, Stottman RW, Brugmann SA (2015). The ciliary baton: Orchestrating neural crest cell development. Curr Top Dev Biol.

[CR10] Clement CA (2013). TGF-B signaling is associated with endocytosis at the pocket region of the primary cilium. Cell reports.

[CR11] Clement CA et al (2009) The primary cilium coordinates early cardiogenesis and hedgehog signalling in cardiomyocyte differenciation. *J. Cell. Science. *3070–3082 10.1242/jcs.049676 PMC2729259 19654211

[CR12] Davis EE, Brueckner M, Katsanis N (2006). The emerging complexity of the vertebrate cilium: New functional roles for an ancient organelle. Dev Cell.

[CR13] Drake JL (2020). How corals made rocks through the ages. Glob Change Biol.

[CR14] Eley L, Yates LM, Goodship JA (2005). Cilia and disease. Curr Opin Genet Dev.

[CR15] Eppard RA, Highison GJ, Mead RW (1989). Scanning electron microscopy of epithelial surfaces of the sea anemone Acontiophorum niveum (Phylumenidaria): Class Anthozoa. J Morphol.

[CR16] Fabbri L (2020). Identification of a new aggressive axis driven by ciliogenesis and absence of VDAC1-DeltaC in clear cell renal cell carcinoma patients. Theranostics.

[CR17] Fautin DG, Mariscal RN (1991) Cnidaria: Anthozoa. *In* Placozoa, Porifera, Cnidaria, and Ctenophora. vol. 2. F.W.H.a.J.A. Westfall, editor. Wiley-Liss, New York. 267–358

[CR18] Ganot P (2020). Ubiquitous macropinocytosis in anthozoans eLife.

[CR19] Gattuso JP, Hansson L (2011) Ocean Acidification. Oxford University Press. pp 313

[CR20] Harii S, Yasuda N, Rodriguez-Lanetty M, Irie T, Hidaka M (2009). Onset of symbiosis and distribution patterns of symbiotic dinoflagellates in the larvae of scleractinian corals. Mar Biol.

[CR21] Johnston IS (1980). The ultrastructure of skeletogenesis in zooxanthellate corals. Int Rev Cytol.

[CR22] Lattao R, Kovács L, Glover DM (2017). The centrioles, centrosomes, basal bodies, and cilia of Drosophila melanogaster. Genetics.

[CR23] Liem KF (2012). The IFT-A complex regulates Shh signaling through cilia structure and membrane protein trafficking. J Cell Biol.

[CR24] Lindbaek L (2015). Coordination of TGFB/BMP signaling is associated with the primary cilium. Cilia.

[CR25] Martin GG (1978). Ciliary gliding in lower invertebrates Zoomorphologie.

[CR26] Mass T (2017). Amorphous calcium carbonate particles form coral skeletons. Proc Natl Acad Sci.

[CR27] Monty JA, Gilliam DS, Banks K, Stout DK, Dodge RE (2006) Coral of opportunity survivorship and the use of coral nurseries in coral reef restoration. *Marine and Environmental Sciences Faculty Proceedings*. 31

[CR28] Moore ER, Jacobs CR (2018). The primary cilium as a signaling nexus for growth plate function and subsequent skeletal development. J Orthop Res.

[CR29] Muscatine L, Tambutté E, Allemand D (1997). Morphology of coral desmocytes, cells that anchor the calicoblastic epithelium to the skeleton. Coral Reefs.

[CR30] Nguyen AM, Jacobs CR (2013). Emerging role of primary cilia as mechanosensors in osteocytes. Bone.

[CR31] Nigg EA, Stearns T (2011). The centrosome cycle: Centriole biogenesis, duplication and inherent asymmetries. Nat Cell Biol.

[CR32] Olsen BR (2005). From the editor's desk. Matrix Biol.

[CR33] Pacherres CO, Ahmerkamp S, Schmidt-Grieb GM, Holtappels M, Richter C (2020). Ciliary vortex flows and oxygen dynamics in the coral boundary layer. Scientific Reports.

[CR34] Pazour GJ, Witma GB (2003). The vertebrate primary cilium is a sensory organelle. Curr Opin.

[CR35] Pitaval A, Tseng Q, Bornens M, Théry M (2010). Cell shape and contractility regulate ciliogenesis in cell cycle–arrested cells. J Cell Biol.

[CR36] Prodromou NV (2012). Heat shock induces rapid resorption of primary cilia. J Cell Science.

[CR37] Puverel S (2005). Antibodies against the organic matrix in scleractinians: A new tool to study coral biomineralization. Coral Reefs.

[CR38] Raz-Bahat M, Erez J, Rinkevich B (2006). In vivo light-microscopic documentation for primary calcification processes in the hermatypic coral *Stylophora pistillata*. Cell Tissue Res.

[CR39] Riisgard HU, Larsen PS (2001). Minireview: Ciliary filter feeding and bio-fluid mechanics- present understanding and unsolved problems. Limnol Oceanogr.

[CR40] Riisgård HU, Larsen PS (2010). Particle capture mechanisms in suspension-feeding invertebrates. Mar Ecol Prog Ser.

[CR41] Riparbelli MG, Persico V, Dallai R, Callaini G (2020). Centrioles and ciliary structures during male gametogenesis in hexapoda: Discovery of new models. Cells.

[CR42] Sevilgen DS et al (2019) Full in vivo characterization of carbonate chemistry at the site of calcification in corals. *Science Advances*. 5:eaau7447. 10.1126/sciadv.aau7447 PMC6357752 30746460

[CR43] Shapiro OH (2014). Vortical ciliary flows actively enhance mass transport in reef corals. PNAS.

[CR44] Singla V, Reiter JF (2006). The primary cilium as the cell's antenna: Signaling at a sensory organelle. Science.

[CR45] Song DK, Choi JH, Kim M-S (2018). Primary cilia as a signaling platform for control of energy metabolism. Diabetes Metab J.

[CR46] Song Z, Zhang X, Jia S, Yelick PC, Zhao C (2016). Zebrafish as a model for human ciliopathies. Journal of Genetics and Genomics.

[CR47] Tambutté SM et al (2011) Coral biomineralization: From the gene to the environment. *J. Exp. Mar. Biol. Ecol. *58–78

[CR48] Temiyasathit S, Jacobs CR (2010). The osteocyte primary cilium and its role in bone mechanotransduction. Ann N Y Acad Sci.

[CR49] Venn AA, Tambutté E, Holcomb M, Allemand D, Tambutte S (2011). Live tissue imaging shows reef corals elevate pH under their calcifying tissue relative to seawater. PLoS ONE.

[CR50] Ware SM, Gunay-Aygun M, Hildebrandt F (2011). Spectrum of clinical diseases caused by disorders of primary cilia. Proc Am Thorac Soc.

[CR51] Yuan X, Serra RA, Yang S (2015). Function and regulation of primary cilia and intraflagellar transport proteins in the skeleton. Ann N Y Acad Sci.

[CR52] Zoccola D (2015). Bicarbonate transporters in corals point towards a key step in the evolution of cnidarian calcification. Sci Rep.

[CR53] Zoccola D (2009). Specific expression of BMP2/4 ortholog in biomineralizing tissues of corals and action on mouse BMP receptor. Mar Biotechnol.

[CR54] Zoccola D (2004). Molecular cloning and localization of a PMCA P-type calcium ATPase from the coral *Stylophora pistillata*. Biochim Biophys Acta.

[CR55] Zoccola D (1999). Cloning of a calcium channel a1 subunit from the reef-building coral, Stylophora pistillata. Gene.

