# Supplementary figures and images for: A role for primary cilia in coral calcification?
Source: Cell Tissue Res. 2020 Dec 16;383(3):1093–102. doi: 10.1007/s00441-020-03343-1 (PMC7960582; doi:10.1007/s00441-020-03343-1)

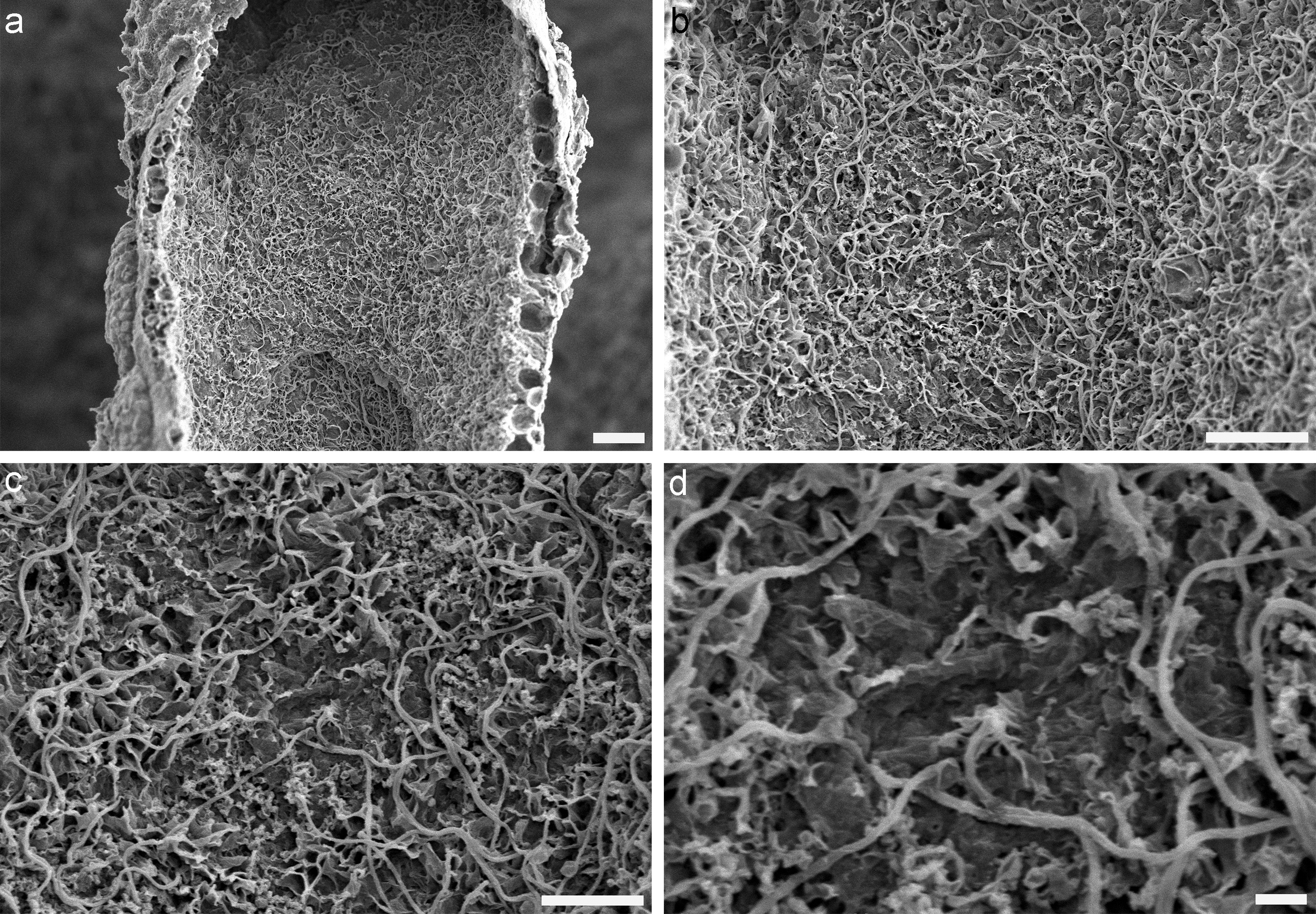

Supplement: Supplementary file 2 — Supplementary file2 (TIF 4.6 kb) [file 441_2020_3343_MOESM2_ESM.tif]

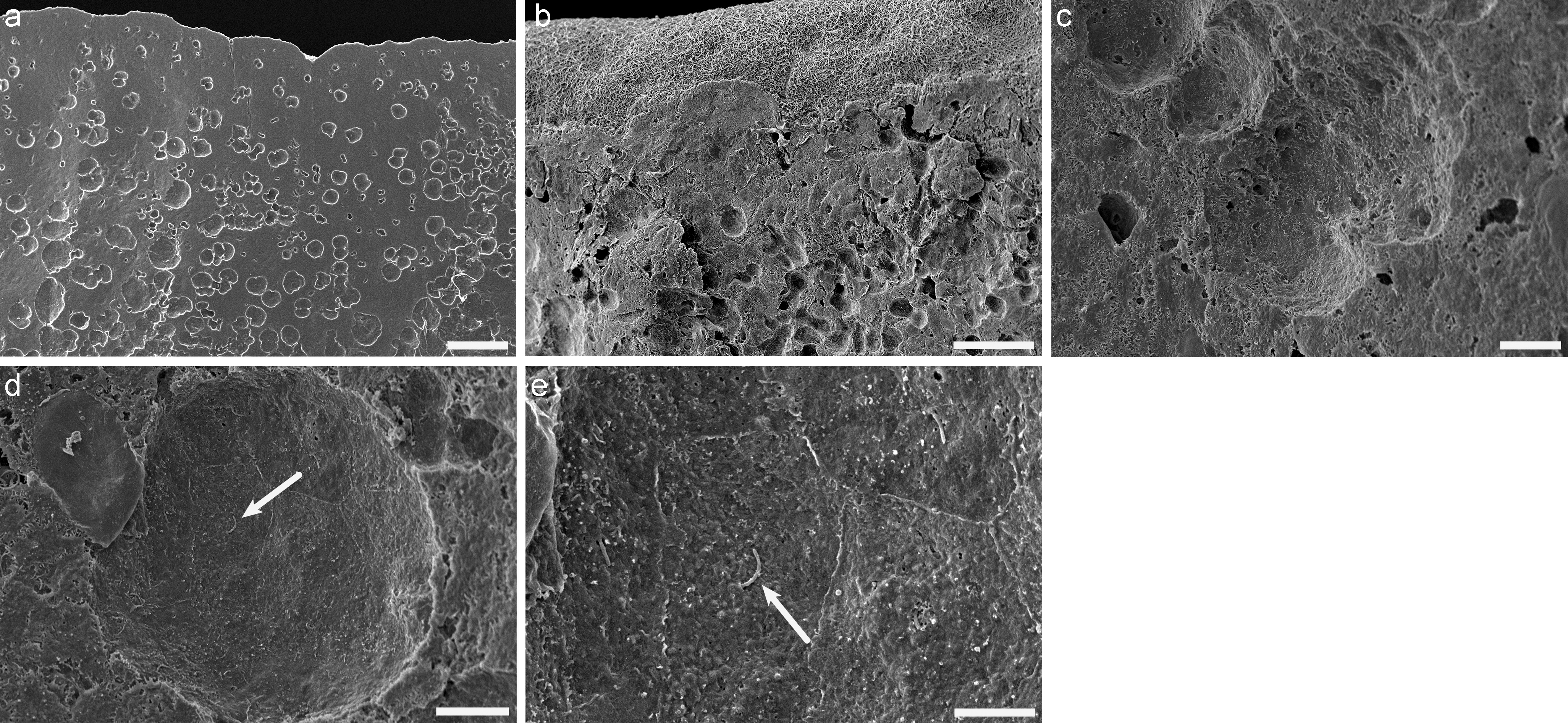

Supplement: Supplementary file 3 — Supplementary file3 (TIF 4.8 kb) [file 441_2020_3343_MOESM3_ESM.tif]
